# Toxic effects of glibenclamide in fetuses of normoglycemic rats: an alternative therapy for gestational diabetes mellitus

**Published:** 2014-05-24

**Authors:** L. Aguillar-Gomes, C.M. Lopes, D.S. Barbieri, T. Rocha, P. Randazzo-Moura

**Affiliations:** 1 *Universidade São Francisco, Avenida São Francisco de Assis, 218, Jardim São José, Bragança Paulista, 12916-900 SP, Brazil*; 2 *Pontifícia Universidade Católica, Rua Joubert Wey, 290, Sorocaba, 18030-070 SP, Brazil*

**Keywords:** Gestational diabetes mellitus, Glibenclamide, Toxicity

## Abstract

Gestational diabetes mellitus (GDM) is defined as glucose intolerance first diagnosed during the second or third trimester of pregnancy. The treatment aims at glycemic control through changes in the patient’s diet with or without exercise, but some patients need insulin therapy. An alternative would be to use oral hypoglycemic agents such as glibenclamide (GLIB). The present study aims to analyze the toxic effects of GLIB in fetuses of pregnant rats which received 5 or 20mg/kg doses of GLIB. Glycemic dosage reveals no significant difference between control (deionized water) and treated groups, showing that these concentrations of GLIB were not effective to cause hypoglycemia in rats. The vitality of the fetuses in all groups was 100%. GLIB administration promoted increase in weight and significant changes in measures of external morphological parameters of treated fetuses. Histological analysis revealed that liver lobes, lobules and central lobular veins were well defined for all treatments. However, GLIB animals presented a light brownish precipitate into the center-lobular veins and in the liver parenchyma among the hepatocytes. These results indicated a possible passage of the drug through the blood-placental membrane, without serious changes that impair the development of neither bone tissue, nor the liver of these animals.

## Introduction

Gestational diabetes mellitus (GDM) is a disease that can affect women during pregnancy. It is defined as glucose intolerance of variable degree with onset or first diagnosis during the second or third trimester of pregnancy (Rotondo and Coustan, 1996; Maganha *et al.*, 2003).

During its development, maternal hyperglycemia, which reaches the fetuses by facilitated diffusion (Aguilar-Bryan *et al.*, 1995), stimulates overproduction of insulin that interferes with fetal homeostasis, might leading to macrosomia, trauma of the birth canal, twisted shoulder, hypoglycemia, hyperbilirubinemia, hypocalcaemia and polycythemia fetal, neonatal respiratory disorders, blindness, kidney failure, and intrauterine fetal death (O`Sullivan *et al.*, 1973; Larimore and Petrie, 2000).

The GDM treatment aims at glycemic control through changes in the patient’s diet with or without exercise. Approximately 15 to 60% of women with GDM need insulin therapy (Bertini, 2000; Volpato *et al.*, 2006). However insulin treatment is expensive, unwieldy and difficult to adhere.

An alternative would be to use oral hypoglycemic agents such as the glibenclamide (GLIB), a second generation sulfonylurea, for Diabetes mellitus treatment. GLIB binds to the SUR1 subunit of K^+^ channel sensitive to ATP and blocks the channel (Aguilar-Bryan *et al.*, 1995). The reduction in K^+^ conductance leads to membrane depolarization and Ca^2+^ influx through voltage sensitive Ca^2+^ channels. The intracellular Ca^2+^ acts as an insulin secretagogue (Aguilar-Bryan *et al.*, 1995) thereby producing hypoglycemic action by increasing insulin release from pancreatic β cells (Nery *et al.*, 2008).

Although some drugs, such as oral hypoglycemic agents, are responsible for 4-5% of fetal malformations (Luiz *et al.*, 2011), studies with model human placenta showed that the transfer of GLIB through the placental barrier is negligible, and the fetuses’ drug concentration does not exceed more than 1-2% of maternal concentration (Albuquerque, 2009).

Given the importance of using an oral hypoglycemic agent in the treatment of GDM, and the need for more information on this drugs’ family (Luiz *et al.*, 2011), the present research aims to study the toxic effects of GLIB in fetuses of pregnant rats which received different doses of GLIB.

## Materials and Methods

All experiments were performed in accordance with standards established by the Brazilian Society of Laboratory Animal Science (SBCAL-old COBEA). The reagents and salts used were obtained from Sigma-Aldrich Chemicals laboratories, Synth, Merck and Bio Rad. Three female Wistar rats (weighing 180 to 200g) were kept with one male for approximately 12 hours. The pregnancy was confirmed by the presence of sperm in vaginal lavage obtained with saline introduced into the vagina, indicating the first day of pregnancy. The pregnant rats were separated from others in order to receive the drug.

Glibenclamide powder (Pharmaceutical Laboratory Galena - Lot: GC/002/4023) (GLIB) was prepared at the time of use, dissolved in 5 drops ethanol in 100 mL of deionized water. The concentrations of 5 and 20mg/kg were administered by oral gavage in the 1st, 5th, 10th and 15th day of gestation. The same protocol was used for control animals which received only deionized water. Rats from all groups were weighed in order to determine the volume to be administered.

Glucose dosages of pregnant animals were determined before GLIB administration and 30 minutes after using glucometer strips (MediSense Optium Xceed, Abbott). The feed intake and weight of pregnant rats were evaluated in the 1st, 5th, 10th, 15th and 18th day, using a semi analytical balance.

After 18-19 days of pregnancy, cesarean sections were performed. The corpora lutea were carefully dissected for subsequent counting, and fetuses were removed with their placentas. After this procedure, the rats were sacrificed by deepening the anesthesia. The following parameters were analyzed: number of corpora lutea and implantations, number of live fetuses, weights of fetuses and corresponding placentas.

The fertility rate of rats was assessed by the following equations:

Pre-implantation loss = (number of corpora lutea – number of deployments) / number of corpora lutea.

Post-implantation loss = (number of deployments – number of living fetuses) / number of deployments.

Other parameters were analyzed as deployment of eyes and ears, limbs, anal drilling, girth, head injury, length and configuration of the tail and toes.

Index vitality was obtained using the following equation:

Number of living and lifeless ____ 100 %

Number of living fetuses ________ × %

After this macroscopic inspection, the fetuses were euthanized by halothane inhalation, weighted and fixed in Bouin solution or diaphanized.

Anatomic measurements were performed using a caliper: anteroposterior skull, lateral-lateral skull, anteroposterior chest, lateral-lateral thorax, craniocaudal and tail, to investigate possible macrosomic changes. The results were represented by the average ± standard deviation. Student t-test was used to compare the means. The significance level was set at p<0.05.

Diaphanized fetuses (xylene and alizarin 2% in 1% KOH solution) were analyzed using a stereoscope to record abnormalities and possible variations in the ossification process. The results were compared to the illustrations of Damasceno *et al*. (2008). The remaining fetuses were fixed in Bouin, dehydrated in crescent series of ethanol, diaphanized in xylene and included in paraffin.

The histological sections (5μm) were deparaffinized in xylene, rehydrated in decreasing ethanol series and stained with hematoxylin-eosin (HE). The slides were individually analyzed and the number of liver center-lobular veins, partially or completely obstructed, was counted in attempting to ascertain the possible cytotoxicity of GLIB. The results were represented by the average of experiments ± standard deviation. One-way ANOVA was used to compare the results. The significance level was p<0.001. Tukey’s Multiple Comparison Test was also applied.

## Results

Glycemic dosage, previously or subsequently to GLIB administration, revealed no significant difference between control and treated groups (p> 0.05), showing that these concentrations of GLIB were not effective to cause hypoglycemia in rats. The glycemic dosage of pregnant Wistar rats pre-treatment and post-treatment (30 minutes after GLIB administration) dosage was less than 120mg/dL in all groups (data not shown).

In relation to feed intake, the results were variable throughout the gestational period, but the rats of control group had a lower intake of feed throughout the gestational period compared to the rats of treated groups.

Regarding the weight gain, the values were variable throughout the gestational period, but after the 15th day of pregnancy, weight gain for treated animals was significantly different when compared to control. The rats treated with 20mg/kg GLIB showed significant increase in weight gain also after the 5th day of gestation (data not shown).

The vitality of the fetuses in all groups was 100%. The pre-implantation loss in the treated groups was not significantly different from the control group. In all groups, absorptions with or without fetal placentas, were observed: 1 reabsorption in the control, 4 reabsorptions in the group treated with GLIB 5mg/Kg and 1 reabsorption in the group treated with 20mg/kg GLIB (data not shown).

**Fig. 1:**
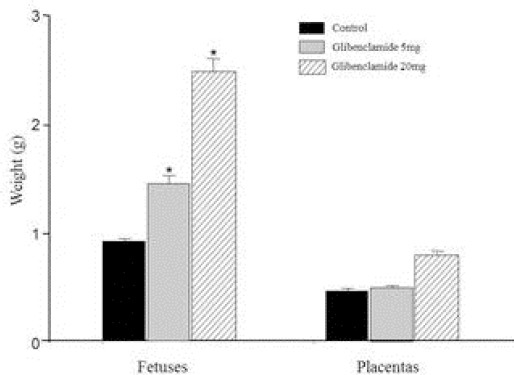
Average-weights of placentas and fetuses of pregnant rats exposed to 0.5mL/Kg of deionized water (control, n = 53), 5mg/Kg GLIB (n = 47) and 20mg/Kg GLIB (n = 60). *p <0.05 compared to the control group.

Regarding fetuses and placentas from animals of group GLIB 20mg/kg the weight was 2.5 ± 0.04 g and 0.79 ± 0.02 g, respectively (Fig. 1).

**Fig. 2:**
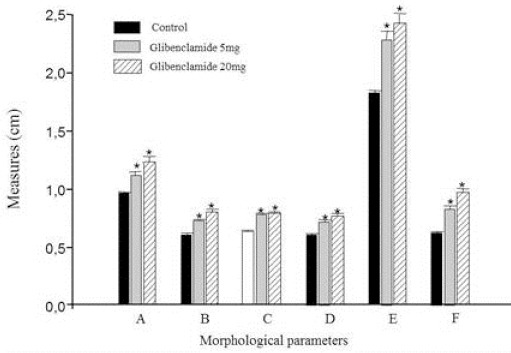
Morphological parameters of fetuses of pregnant rats exposed to 0.5mL/Kg of deionized water (control, n = 26), 5mg/Kg GLIB (n = 23) and 20mg/Kg GLIB (n = 30). The panel represents the measurements performed: anteroposterior (A) and lateral-lateral (B) skull, anteroposterior (C) and lateral-lateral (D) of the chest, head-tail (E) and tail (F). * p<0.05 when compared to the control group.

**Fig. 3:**
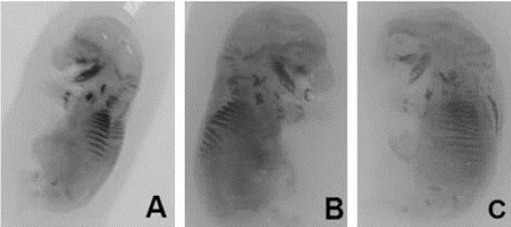
Diaphanized fetuses. (A) Control group, (B) 5mg/Kg GLIB group, (C) 20mg/Kg GLIB group.

Considering the external morphological parameters, 5 and 20mg/kg GLIB administration promoted significant changes in measures when compared to the control group (Fig. 2-3).

**Fig. 4:**
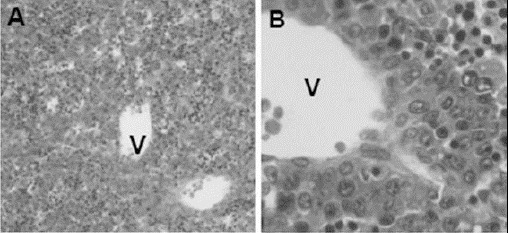
Histological features of the fetuses livers of pregnant rats exposed to 0.5mL/Kg of deionized water (control, n = 3) (A - 100x, B - 400x). V = central vein.

**Fig. 5:**
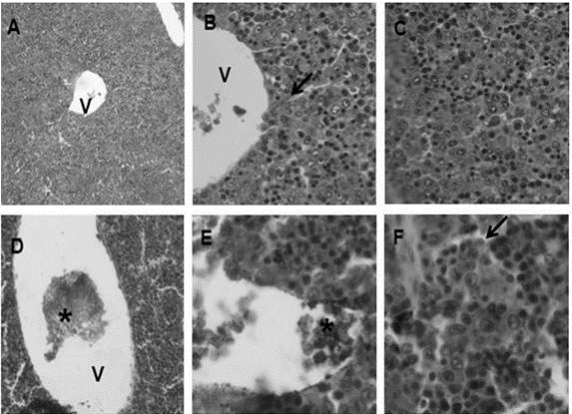
Histological features of fetuses livers of pregnant rats exposed to5mg/Kg GLIB (A, B, C) and 20mg/Kg GLIB (D, E, F). Note the presence of a brownish precipitate (*) in the lumen of the central vein (V) and in the liver parenchyma. Arrow = capillary sinusoid. A, D: 100x, B, C, E, F: 400x.

Histological analysis of median sagittal section revealed that liver lobes, lobules and central lobular vein were well defined for all treatments. However, animals treated with GLIB presented a light brownish precipitate into the center-lobular veins (partially or totally) and in the liver parenchyma among the hepatocytes (Fig. 4-5).

The control animals showed 100% of the center-lobular veins free of precipitate, while the treated GLIB 5mg/Kg group showed a predominance of center-lobular veins without precipitate and only 9% (corresponding to 3 veins) of the total center-lobular veins with precipitate. Unlike GLIB 20mg/kg group presented a light brown precipitate in 43% (corresponding to 24 veins) of total veins.

## Discussion

Considering the incidence and severity of GDM, the study of a new drug therapy became necessary. For these reasons GLIB, a second-generation sulfonylurea, has been shown as an option, considering its low permeability throw hematoplacental barrier (Silva *et al.*, 2007b).

According to Aberg *et al*. (2001), glycemic control is particularly important to reduce perinatal complications (Aberg *et al.*, 2001; Valdés *et al.*, 2008). Comparative studies of glycemic control using GLIB and insulin showed that 18.75% of pregnant women have not achieved glycemic control with GLIB (Silva *et al.*, 2007a). A study with 50 pregnant women with GDM and treated with glyburide showed 58% efficacy (Silva *et al.*, 2007b). However the success rate of using glibenclamide varies greatly among studies, probably due to the use of different doses and protocols (Langer, 2002).

The effectiveness of GLIB on glycemic control could be evaluated using glucose measurements before and after drug administration. The present study showed no significant differences between treated and control animals, indicating that the studied doses were not able to change the normoglycemia of pregnant rats (Valdés *et al.*, 2008).

The weight gain of animals exposed to any chemical agents during a specific period is a parameter commonly used to determine their toxic effects (O`Sullivan *et al.*, 1973). Experiments using 20mg/kg showed weight gain, possibly as a compensatory mechanism for hypoglycemic, suggesting that this dose was able to increase insulin secretion, decrease hepatic production of glucose, resulting in reversal of hyperglycemia and, indirectly, in increased insulin sensitivity in tissues of normoglycemic individuals, simulating a fast. Consequently the hunger center can be activated and the individual tends to feed unnecessarily and excessively, leading to weight gain (Guyton and Hall, 2006).

Regarding acute toxicity (Hellmuth *et al.*, 1994; Larimore and Petrie, 2000; Albuquerque, 2009), reproductive capacity and physical development of rat fetuses (Albuquerque, 2009), the pre-and post-implantation blastocyst undergoes physiological changes when exposed to chemicals, serving this parameters to distinguish embryotoxicity of direct toxic effects on uterine functions, which can influence fertility, followed by pregnancy prematurely interrupted, and possible fetal malformations (Khera, 1984; Gerenutti *et al.*, 2006). Results of treated groups showed corpora lutea with no corresponding fetuses. The presence of fetal reabsorptions indicates a failure in embryonic development, probably occasioned by drug administration.

The rate of pre-implantation losses establishes the relationship between two variables: number of corpora lutea and number of deployments. The size (or weight) of corpora lutea correlates with the concentration of circulating progesterone (Cumming, 1990), which is one of the major hormones to maintain pregnancy (Uchida *et al.*, 1970).

It is known that the rate of implantation correlates with the number of corpora lutea, and is a success indicator of blastocyst implantation into the endometrium (Kato *et al.*, 1979).

Teratogenicity is characterized by retarded fetal development with reduced fetal weight.

The implantation of the blastocyst in pregnant mammals requires an integrated series of phenomena which include uterine preparation, embryo transportation, embryonic annexes, uterine transformation, placental development and hormonal system to support each step (Alivert *et al.*, 1979; Ford, 1982).

As a fetal organ, the placenta is exposed to the same influences of intrauterine environment and any trauma affects the fetuses (Damasceno *et al.*, 2008). Proper placental functioning is crucial for the development of the fetuses, and the pattern of morphological alteration found may indicate clinical changes related to maternal and fetal intrauterine development.

The placental barrier between mother and fetuses has a protective function, however limited, because many drugs can cross it, either by simple diffusion, facilitated diffusion or active transport (Hagerman and Villee, 1960; Beebe *et al.*, 1996).

The fetuses of treated groups showed macrosomia (p <0.05 compared to control), corroborating to other comparative studies with insulin and GLIB, which also showed macrosomia for GLIB (Hagerman and Villee, 1960).

On the other hand, Langer *et al*. (2000) found no difference between the two groups. Literature data show that maternal weight gain during pregnancy is associated with fetal macrosomia, even in the absence of diabetes mellitus, because obesity induces fetal hyperinsulinemia, increased plasma triglycerides, increased transplacental transport of fatty acids, justifying such morphological changes (Montenegro and Rezende, 2008).

There was no significant increase in placental weight for control and treated groups. Comparing the findings with human placental development, the placental weights in full-term fetuses represent 1/6 of their birth weight. The evolution of the placental weight during pregnancy in humans shows that, initially, the weight exceeds the conceptus, equaling around 14 weeks. So over the course of pregnancy the placenta undergoes involution, which is not a good parameter to evaluate embryonic development during late gestation (Montenegro and Rezende, 2008).

Fetuses undergoing the evisceration and clarifying techniques showed that macroscopic and qualitative parameters of bone marrow remained unchanged in all experimental groups. Histological analysis of GLIB 5 and 20mg/kg showed that there is no alteration in the fetuses liver histoarchitecture, but revealed the presence of a light brownish precipitate in the central-lobular vein, as well as in the liver parenchyma among the hepatocytes.

This precipitate resembles glycoprotein expression on the surface membrane of liver hepatocyte in rats submitted to polychemotherapy, suggesting a possible toxicity of glibenclamide (Sen`kova *et al.*, 2012). These results indicated a possible passage of the drug through the blood-placental membrane, observed as a light brownish precipitate into the central-vein of the liver lobules. However, no serious anatomical changes that impair the development of bone tissue, or the anatomy and histology of the liver were observed in these animals.

## References

[ref1] Aberg A, Rydhstrom H, Frid A (2001). Imparied glucose tolerance associated with adverse pregnancy outcome: a population-based study in southern Sweden. Am. J. Obstet. Gynecol.

[ref2] Aguilar-Bryan L, Nichols C.G, Wechsler S.W, Clement J.P, Boyd A.E, González G, Herrera-Sousa H, Nguy K, Bryan J, Nelson D.A (1995). Cloning of the beta cell high-affinity sulfonylurea receptor: A regulator of insulin secretion. Science.

[ref3] Albuquerque L.B.L (2009). Estudos *in vitro* e *in vivo* da Plathymenia reticulata Benth. [dissertação].

[ref4] Alivert V, Bonanomi L, Giavini E, Leone V.G, Mariani L (1979). The extent of fetal ossification as an index of delayed development in teratogenic studies on the rat. Teratology.

[ref5] Beebe L.A, Cowan L.D, Altshuler G (1996). The epidemiology f placental features: associations with gestacional age and neonatal outcome. Obstet. Gynecol.

[ref6] Bertini A.M (2000). Diabetes mellitus e gravidez. Federação Brasileira de Ginecologia e Obstetrícia. Tratado de obstetrícia.

[ref7] Cumming A.M (1990). Toxicological mechanisms of implantation failure. Fundam. Appl. Toxicol.

[ref8] Damasceno D.C, Kempinas W.G, Volpato G.T, Consoni M, Rudge M.V.C, Paumgartten F.J.R (2008). Anomalias congênitas: estudos experimentais.

[ref9] Ford W.C.L (1982). The effect of deoxy-6-fluoroglucose on the fertility of male rats and mice. Contraception.

[ref10] Gerenutti M, Del Fiol F, Groppo F.C (2006). Performance reprodutiva de ratas grávidas e efeitos embriotóxicos da ciprofloxacina. Pharmazie.

[ref11] Guyton A.C, Hall J.E (2006). Tratado de Fisiologia Médica.

[ref12] Hagerman D.D, Villee C.A (1960). Transport functions of the placenta. Physiol. Rev.

[ref13] Hellmuth E, Damm P, Molsted-Pedersen L (1994). Congenital malformations in offspring of diabetic women treated with oral hypoglycemic agents during embryogenesis. Diabet. Med.

[ref14] Kato H, Morishige W.K, Rotchild I (1979). A quantitative relationship between the experimentally determined number of conceptuses and corpus luteum activity in pregnant rat. Endocrinology.

[ref15] Khera K.S (1984). Maternal toxicity - a possible factor in fetal malformations in mice. Teratology.

[ref16] Langer O (2002). Oral hypoglycemic agents and pregnant diabetic: “from bench to bedside”. Semin Perinatol.

[ref17] Langer O, Conway D.L, Berkus M.D, Xenakis E.M.J, Gonzales O (2000). A comparison of glyburide and insulin in women with gestacional diabetes mellitus. N. Engl. J. Med.

[ref18] Larimore W.L, Petrie K.A (2000). Drug use during pregnancy and lactation. Update in maternity care. Prim. Care.

[ref19] Luiz K.J, Maria N.C.K, Marisa P.Q, Mary U.N, João A.V.M, Briquet R, Guariento A (2011). Farmacocinética, metabolismo e transferência de drogas. Obstetrícia normal.

[ref20] Maganha C.A, Vanni D.G.B.S, Bernardini M.A, Zugaib M (2003). Tratamento do diabetes melito gestacional. Rev. Assoc. Med. Bras.

[ref21] Montenegro C.A.B, Rezende Filho J (2008). Obstetrícia Fundamental.

[ref22] Nery C.G.C, Pires M.A.S, Pianetti G.A, Vianna-Soares C.D (2008). Caracterização do fármaco hipoglicemiante glibenclamida. Rev. Bras. Ciênc. Farm.

[ref23] O`Sullivan J.B, Charles D, Mahan C.M, Dandrow R.V (1973). Gestational diabetes and perinatal mortality rate. Am. J. Obstet. Gynecol.

[ref24] Rotondo L, Coustan D.R, Knuppel RA, Drukker JE (1996). Diabetes mélito na gestação. Alto risco em obstetrícia: um enfoque multidisciplinar.

[ref25] Sen`kova A.V, Mironova N.L, Patutina O.A, Ageeva T.A, Zenkova M.A (2012). The toxic effects of polychemotherapy onto the liver are accelerated by the up regulated MDR of lymphosarcoma. ISRN Oncol.

[ref26] Silva J.C, Bertini A.M, Taborda W, Becker F, Bebber F.R.L, Aquim G.M.D.C, Viesi J.M.Z (2007a). Glibenclamida no tratamento do diabetes melito gestacional em estudo comparado àinsulina. Arq. Bras. Endocrinol. Metab.

[ref27] Silva J.C, Heinen A, Scheidt M.B, Marcondes M.A.O, Bertini A.M (2007b). Tratamento do diabetes mellitus gestacional com glibenclamida - fatores de sucesso e resultados perinatais. Rev. Bras. Ginecol. Obstet.

[ref28] Uchida K, Kadowaki M, Nomura Y, Miyata K, Miyake T (1970). Relationship between ovarian progestin secretion and corpora lútea function in pregnant rat. Endocrinol. Jpn.

[ref29] Valdés R.E, Soto-Chacón E, Lahsen R.M, Barrera C.H, Candia P.P (2008). Eficacia de los hipoglicemiantes orales em el control metabólico de pacientes con diabetes mellitus gestacional. Rev. Med. Chile.

[ref30] Volpato G.T, Damasceno D.C, Campos K.E, Rocha R, Rudge M.V.C, Calderon I.M.P (2006). Avaliação do efeito do exercício físico no metabolismo de ratas diabéticas prenhes. Rev. Bras. Med. Esporte.

